# Supplementary material for: Development of a Clinical–Biological Model to Assess Tumor Progression in Metastatic Pancreatic Cancer: Post Hoc Analysis of the PRODIGE4/ACCORD11 Trial
Source: Cancers (Basel). 2022 Oct 16;14(20):5068. doi: 10.3390/cancers14205068 (PMC9599967; doi:10.3390/cancers14205068)
Supplement: Supplementary file 1 [file cancers-14-05068-s001.zip › cancers-1916121-supplementary.pdf]

Supplementary Table S1. Selected and excluded patients in our study and their overall and progression-free survival.

	**Excluded Patients N=146**	**Selected Patients N=196**	**p-value**
ARM, *no (%)*			0.662
FOLFIRINOX	71(48.6%)	100 (51.0%)	
Gemcitabine	75 (51.4%)	96 (49.0%)	
**At inclusion**			
Sex, *no (%)*			0.119
Men	97 (66.4%)	114 (58.2%)	
Women	49 (33.6%)	82 (41.8%)	
Age (*years*)			0.010
Median	62	59	
Range	31–76	25–75	
ECOG performance status, *no (%)*			0.800
0	52 (36.1%)	75 (38.3%)	
1	94 (64.4%)	120 (61.2%)	
2	0	1 (0.5%)	
Pancreatic tumor location, *no (%)*			0.625
Head	56 (38.4%)	74 (37.8%)	
Body	45 (30.8%)	66 (33.7%)	
Tail	38 (26.0%)	52 (26.5%)	
Multicentric	7 (4.8%)	4 (2.0%)	
No. of metastasis involved			0.976
Median	2	2	
Range	1–6	1–6	
**During follow-up: FOLFIRINOX**			
Overall survival (*months*)			< 0.001
Median	6.8	13.1	
95% CI	4.6 – 9.4	11.7 – 13.7	
Progression-free survival (*months*)			< 0.001
Median	2.8	7.9	
95% CI	1.8 – 4.5	6.9 – 9.0	
**During follow-up: Gemcitabine**			
Overall survival (*months*)			< 0.001
Median	3.8	8.1	
95% CI	3.0 – 6.1	7.2 – 9.3	
Progression-free survival (*months*)			0.003
Median	2.2	3.6	
95% CI	1.8 – 3.1	3.3 – 4.3	

No: number; CI: confidence interval
